# The Physiotherapy Management of Postoperative Mid-shaft Clavicular Fracture With Brachial Plexus Injury: A Case Report

**DOI:** 10.7759/cureus.66461

**Published:** 2024-08-08

**Authors:** Anjali Rai, HV Sharath, Raghumahanti Raghuveer, Moh'd Irshad Qureshi

**Affiliations:** 1 Department of Neuro-Physiotherapy, Ravi Nair Physiotherapy College, Datta Meghe Institute of Higher Education and Research, Wardha, IND

**Keywords:** quality of life, rehabilitation, pseudomeningoceles, epidural collection, brachial plexus injury (bpi)

## Abstract

Traumatic brachial plexus injury (BPI) is a debilitating condition predominantly affecting young males, often resulting from traction or direct injuries. Due to the complicated neuronal network, the damage is often classified as preganglionic or postganglionic injuries. It includes upper-limb mobility impairments as well as reduced muscular strength and sensitivity. We discuss a case of a 30-year-old female who suffered a displaced mid-shaft clavicular fracture and BPI after a road traffic collision. The patient experienced significant pain, restricted movement, and sensory and motor loss in her left arm. Imaging studies revealed additional complications, including epidural collection and pseudomeningoceles. She underwent open reduction and internal fixation of the clavicle, followed by a structured rehabilitation program focusing on pain management, muscle re-education, and functional recovery.

This case highlights the complexity of managing clavicular fractures with concurrent BPI, requiring a multidisciplinary approach involving imaging, surgical intervention, and comprehensive physiotherapy for optimal recovery and functional restoration. Rehabilitation strategies were employed to address the diverse symptoms, including multi-sensory strategies, sensory re-education, graded motor imagery rehabilitation, and gradual restoration of upper extremity (UE) range, strength, and endurance and to develop neuromuscular control. Effective management of clavicular fractures with BPI requires early diagnosis, surgical intervention, and structured rehabilitation to improve functional outcomes and quality of life.

## Introduction

Brachial plexus injury (BPI) is a devastating condition that predominantly impacts young males in their prime working years, resulting in a massive socioeconomic burden [1]. Based on the mechanism of injury, BPI can be classified into two main types: traction injuries leading to avulsion or rupture of cervical roots, and direct injuries affecting the brachial plexus trunks, cords, or nerves [2]. BPI is a rare and complex issue, with very few cases documented in the literature [3]. The patient evaluation involves a detailed examination of motor and sensory functions in the upper extremities (UEs), along with the use of radiological and electrodiagnostic studies. Conservative management tends to be successful, resulting in recovery over several months [4].

Clavicle fractures, often caused by falls, can cause pain and break sound. Physical examination may show edema, soreness, or deformities. Neurovascular exams are crucial due to the close proximity of the subclavian arteries and brachial plexus. A lung examination is also necessary for lung apex damage [5]. In approximately 1-3% of cases involving clavicle fractures, patients may encounter acute complications. These complications often manifest as neurovascular injuries, encompassing damage to the subclavian vein and/or artery injury, brachial plexus, thrombosis, or stenosis [6]. Additionally, lung injuries or pneumothorax, as well as related musculoskeletal injuries, are also observed as potential complications [7].

BPI is a prevalent condition among males aged 15-25 years, with traffic accidents accounting for 70% of traumatic BPI cases. These injuries often involve multiple damages, such as supraclavicular plexus lesions, lower plexus root avulsions, and chronic pain [8]. The second most common cause of BPI is ballistic trauma, which leads to neuropraxia. Such injuries significantly impair daily activities and result in long-lasting debility. Both the patient and their caregivers may experience psychosocial and socioeconomic burdens.

The spinal nerves are formed by the convergence of dorsal and ventral rootlets from the spinal cord, and injuries occurring before the dorsal root ganglion are called "preganglionic," while those occurring after it are termed "postganglionic." Differentiating between preganglionic and postganglionic BPIs is key for determining the appropriate treatment and prognosis. Early surgical intervention is necessary for spontaneous recovery after preganglionic injuries [9]. BPI is an uncommon occurrence following adult clavicle fractures, with only a limited number of cases in the medical literature. The majority of these cases involve brachial plexus compression resulting from fracture displacement or hypertrophic callus formation.

The need for rehabilitation following BPI is on the rise as a result of advancements in diagnosis and surgical procedures. Treatment approaches differ based on the intricate nature of the brachial plexus, the location of the injury, and its underlying causes [10]. Neuromuscular electrical stimulation (NMES) is a promising tool for the rehabilitation of peripheral nerve injuries and their functional recovery. However, research is scarce on acute traumatic BPIs in the polytrauma population, particularly in younger males with multiple-site trauma [11].

As Major Trauma Centres continue to expand, the demand for rehabilitation services for polytrauma patients with BPIs outside specialized peripheral nerve centers is on the rise. Unfortunately, there is currently no nationally agreed-upon rehabilitation pathway for complex BPIs in the polytrauma population, potentially compromising their overall function and quality of life [12]. Research is centered on various interventions such as exercise, sensory training, neuroelectromagnetic stimulation, acupuncture, massage therapy, hydrotherapy, phototherapy, and neural stem cell therapy [13].

## Case presentation

Patient information

A 30-year-old female presented to the emergency department following a road traffic collision. The patient reported a single episode of vomiting, neck pain, left shoulder pain, an inability to use her left arm, and complaints of chest injury. Upon admission, she exhibited irritability and transient loss of consciousness with a Glasgow Coma Scale (GCS) score of 10.

Investigation

A CT brain scan was performed immediately, revealing no abnormalities. Radiographs of the chest showed b/l pleural effusion and the left UE identified a displaced mid-shaft clavicular fracture and shoulder subluxation. Before surgery, an MRI was conducted to evaluate potential complications, revealing epidural collection at C4, C5, and C6 suggestive of pseudomeningoceles and preganglionic nerve injury. The patient was subsequently recommended for open reduction and internal fixation of the clavicular fracture. After surgery, nerve conduction velocity testing (NCV) was done, which pointed to a left global brachial plexus lesion at the postganglionic proximal upper trunk and preganglionic C7, C8-T1 root level lesion.

Clinical examination

After obtaining verbal consent, a comprehensive physical examination was conducted as the patient was conscious, cooperative, and well-oriented to time, place, and person. The examination revealed normal vital signs with no other systemic abnormalities. Local examination showed an abrasion on the left side of the chest and a contusion with swelling over the left supraclavicular region. Palpation indicated grade 3 tenderness. The pain was sudden in onset, exacerbated by UE movements, and alleviated by rest and medications. The pain was located at the back of the neck, anterior aspect of the clavicle, and lateral aspect of the arm, with no temporal variations, and was described as dull-aching. The patient also exhibited paraspinal muscle spasms. Her range of motion was painful and incomplete. Sensory examination revealed loss of superficial as well as deep sensation over left arm. Reflex assessment was also done, which is shown in Table 1.

**Table 1:** Reflex evaluation for nerve roots of upper extremity

Reflex	Nerve	Left side
Biceps (C5-C6)	Musculocutaneous	Diminished
Supinator (C5-C6)	Radial	Diminished
Triceps (C7-C8)	Radial	Diminished
Finger flexion (C7-C8)	Median and ulnar nerve	Diminished

Postural examination

With the patient's verbal consent, a comprehensive postural assessment was conducted in three views: anterior, lateral, and posterior.

Anterior and Posterior View

**Figure 1:**
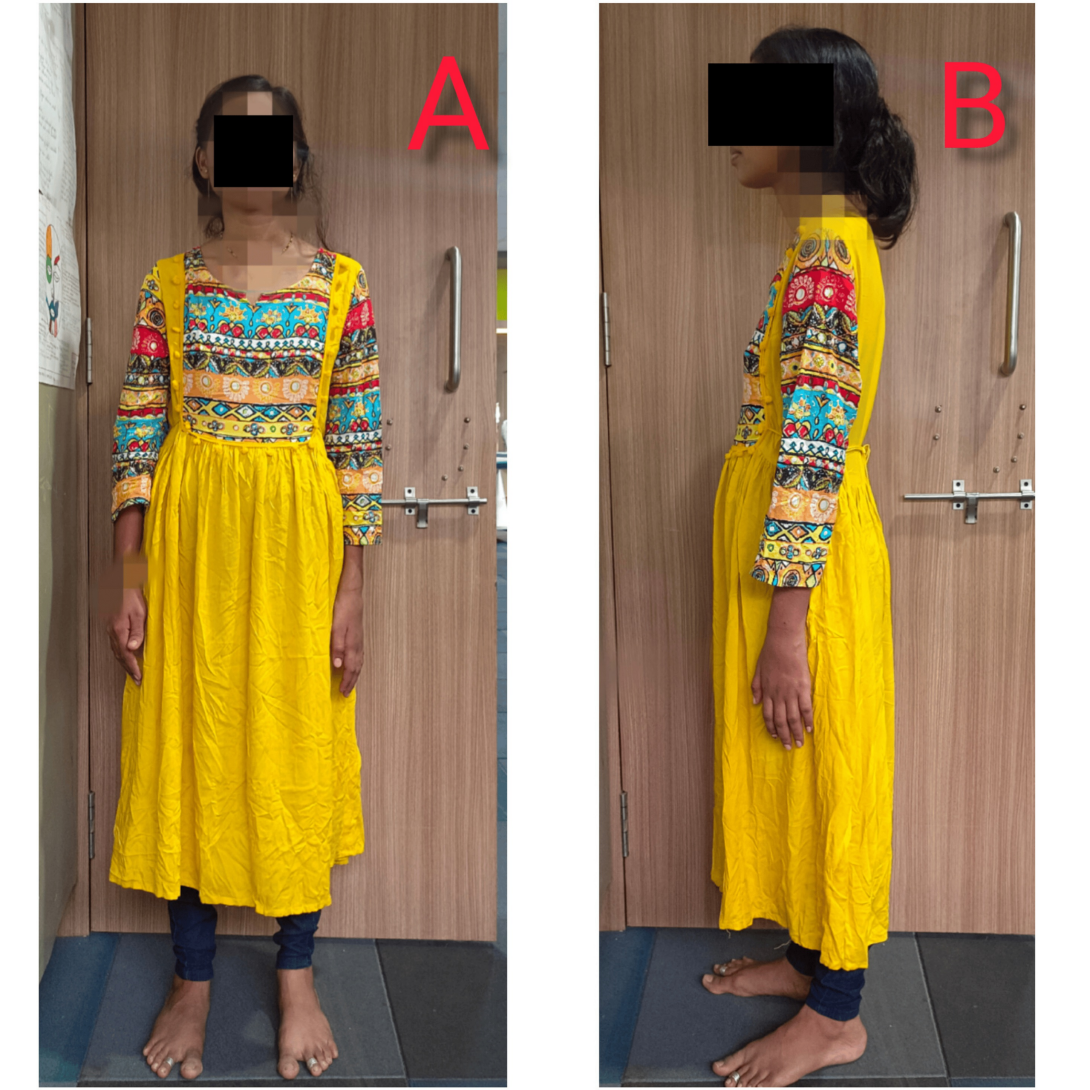
Anterior and lateral views of postural evaluation of the patient A: anterior view evaluation; B: lateral view evaluation

The left shoulder was slightly depressed while the remaining body segments were aligned in a neutral position as shown in Figure 1A. The left shoulder appeared slightly depressed, with a slight dipping of the left pelvis. The right hip exhibited slight internal rotation as shown in Figure 1B.

Lateral View 

**Figure 2:**
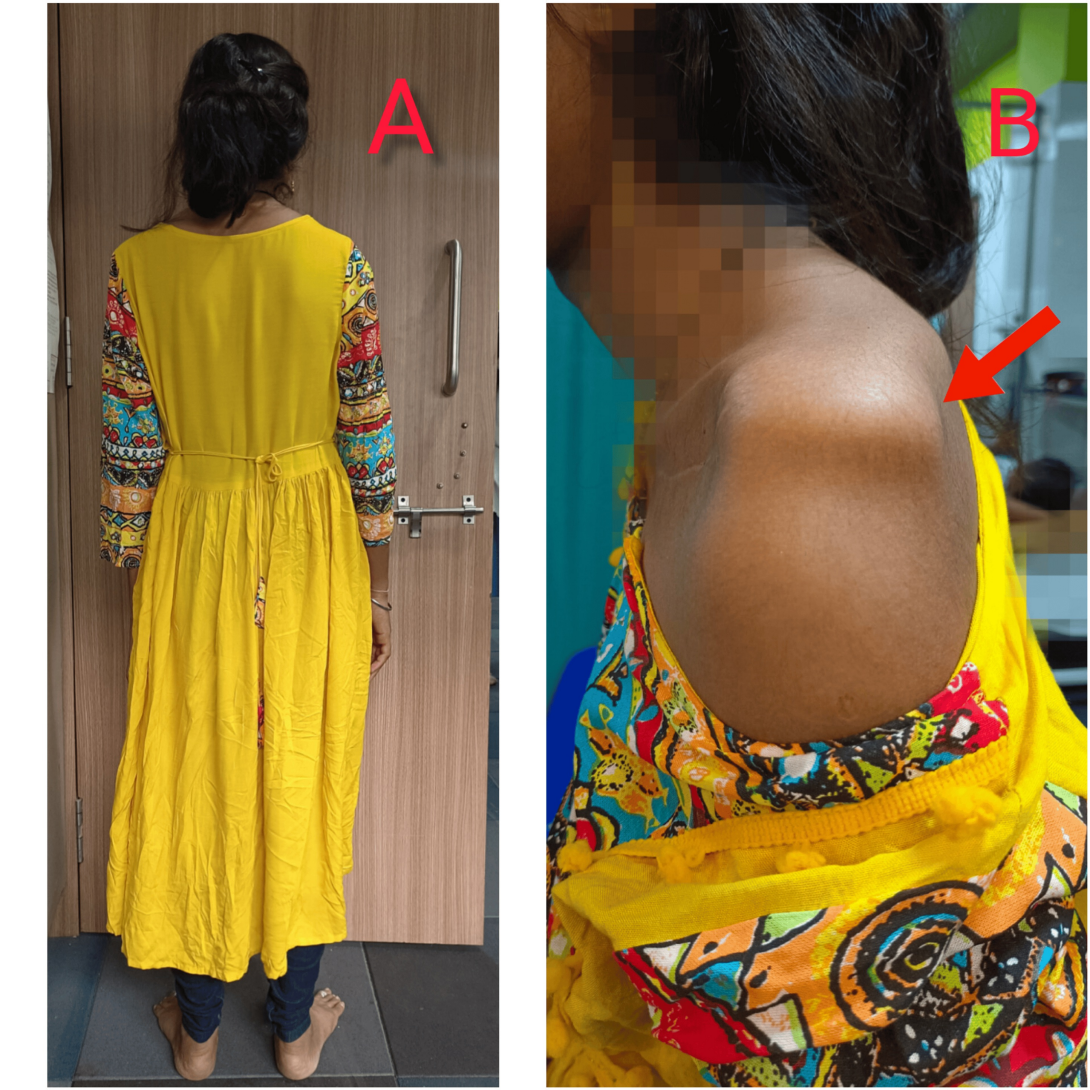
Images showing posterior views of postural evaluation A: posterior view of posture assessment; B: dipping of the right humeral head, i.e., positive sulcus sign is shown

A forward neck posture was observed, along with a positive sulcus sign on the left shoulder. Additionally, a slight anterior pelvic tilt was noted as shown in Figure 2.

Diagnostic assessment

**Figure 3:**
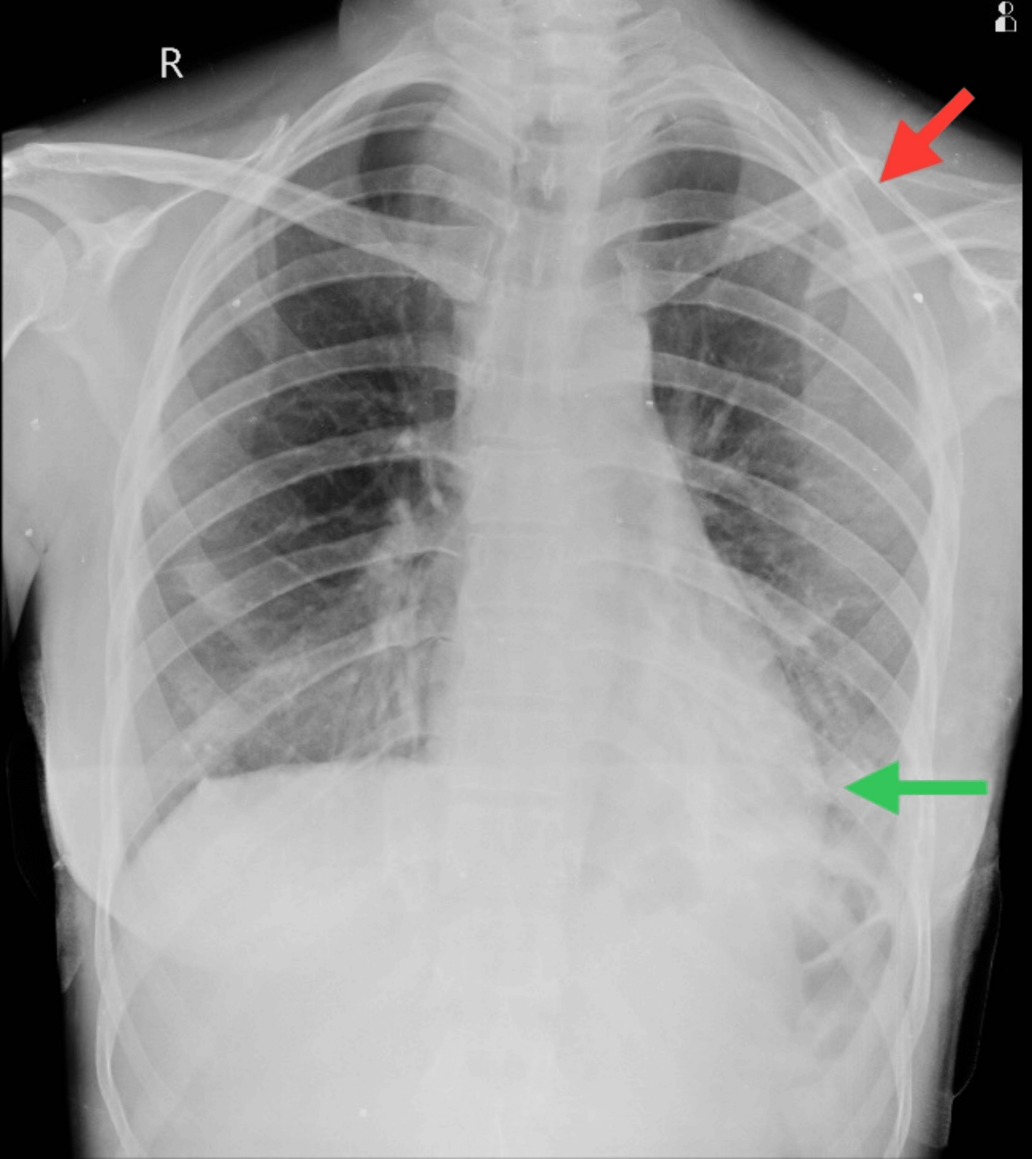
Chest X-ray in the anteroposterior view Red arrow: fracture of mid-shaft 1/3rd of the clavicle which is displaced. Green arrow: mild pleural effusion of the left lower lobe of the chest

**Figure 4:**
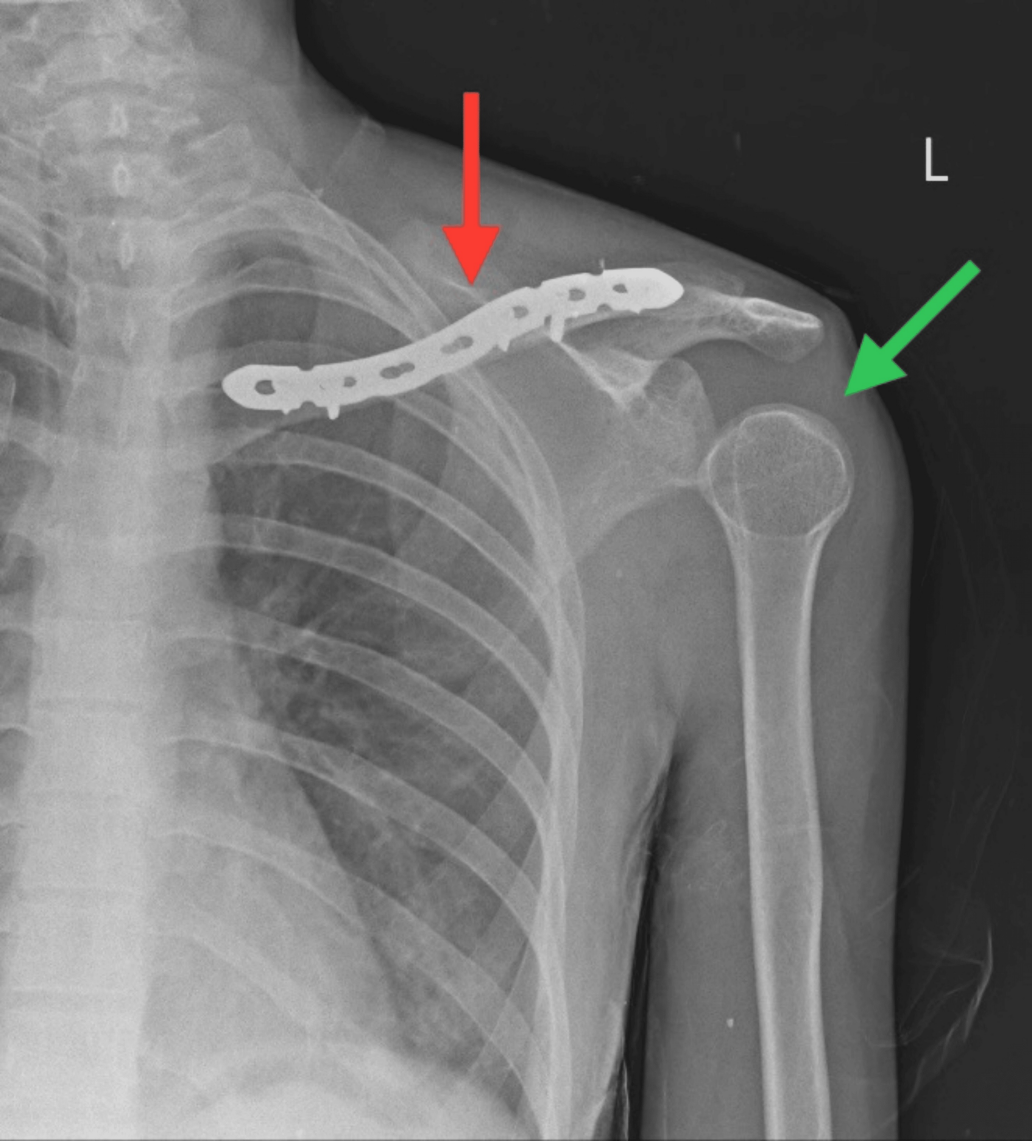
Postoperative radiograph of the left shoulder in the anteroposterior view Red arrow: internal fixation of the clavicle fracture with plate osteosynthesis. Green arrow: subluxation of the left shoulder joint

**Figure 5:**
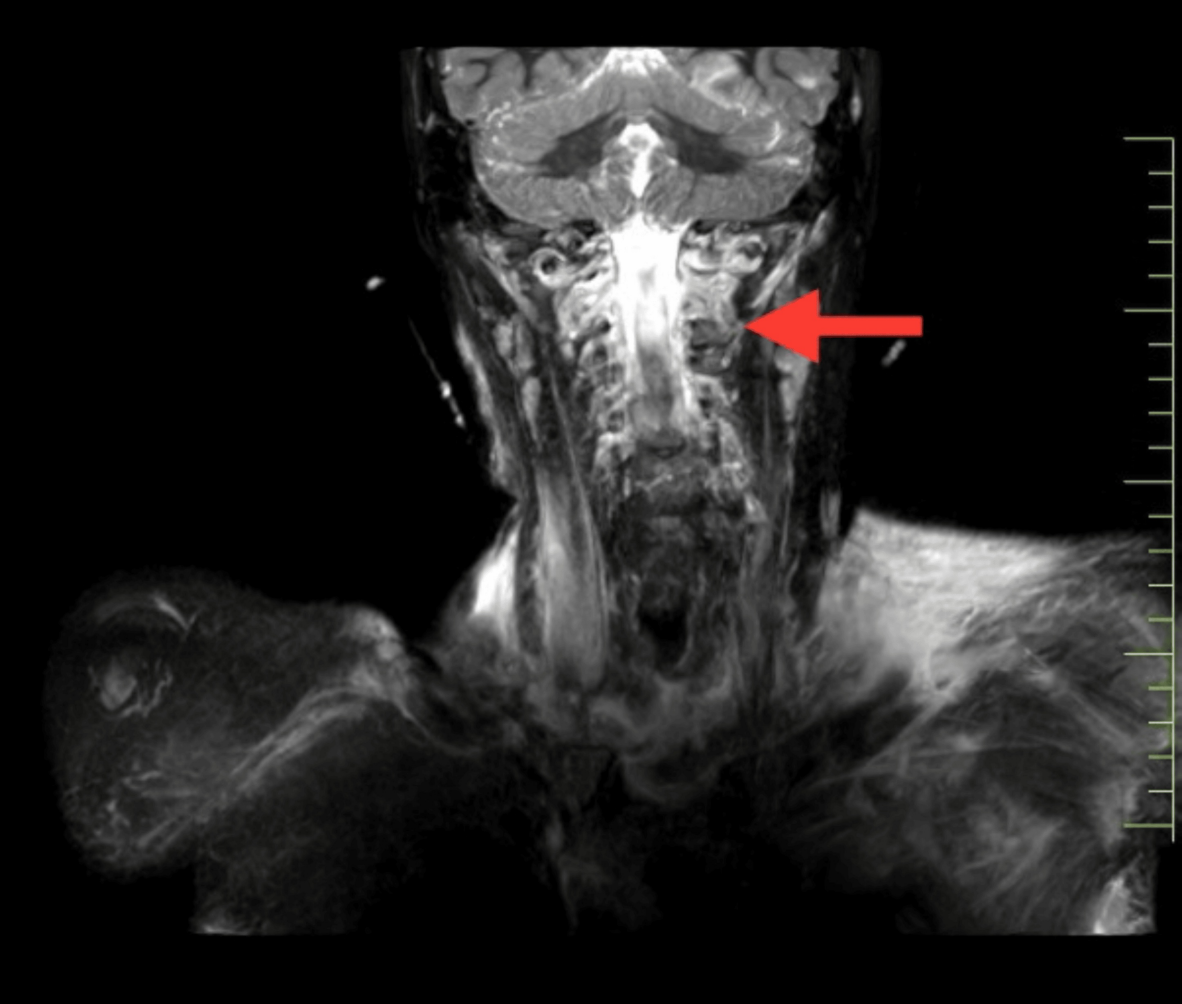
MRI of brachial plexus providing an impression of preganglionic nerve injury MRI: magnetic resonance imaging

The patient underwent a diagnostic assessment, including a chest X-ray that revealed mild blunting of the left costophrenic angle with mild pleural effusion/hemothorax and linear displaced fracture of the middle 1/3rd of the clavicle as shown in Figure 3. Furthermore, a radiograph of the UE was taken postoperatively, showing internal fixation of the fracture with shoulder subluxation as shown in Figure 4. Subsequently, an MRI scan of the cervical spine was conducted, revealing epidural collection at the left side of C4, C5, and C6 levels suggestive of pseudomeningocele, preganglionic nerve injury as shown in Figure 5.

Therapeutic interventions

In phase 1 (zero to four weeks) of the intervention for a patient, proprioceptive neuromuscular facilitation (PNF) techniques are employed to promote muscle strength, flexibility, and coordination. This initial phase focuses on gentle, assisted movements to enhance proprioception and neuromuscular control. The techniques include rhythmic initiation, where movements are guided passively before gradually incorporating active participation, and alternating isometrics, which involve resistance applied in various directions to improve stability. The goal during this phase is to facilitate neuromuscular re-education, reduce muscle stiffness, and lay the groundwork for more intensive therapy in subsequent phases, all the while ensuring the patient's comfort and preventing further injury (Table 2).

**Table 2:** Phase 1 (0-4 weeks) intervention provided to the patient* *[14] PNF: proprioceptive neuromuscular facilitation; NA: not applicable

Goals	Intervention	Rationale	Dosage
To develop strategies to manage and cope with pain or discomfort	Patient and caregiver education	Educate the patient about his condition, potential complications, and preventive strategies, as well as the physiotherapy protocol to be followed	NA
To relieve dyspnea	The Semi-Fowler's position, head end elevated to 45 degrees	Improves breathing by use of gravitational forces to assist in developing the lungs and reduce abdominal pressure on the diaphragm	NA
To enhance breathing and facilitate mobilization	Diaphragmatic breathing	Increase the excursion of the diaphragm and improve gas exchange and oxygenation	Repetitions – 10 times/1 set/every 3-4 hours
To prevent complications of joint contracture and subluxation	Immobilization through sling or hemi-sling/Velpeau bandaging as shown in Figure 6	To prevent joint contracture and uncontrolled limb motion or positions as well as minimize glenohumeral subluxation	Throughout the day
To prevent muscle de-conditioning	Active cervical retraction with the patient sitting with scapulae retracted and unilateral scapular circles	For improving stability and facilitating motor re-education and scapular synergy	10-15 times with 5-seconds hold
To reduce inflammation and edema and to facilitate nerve regeneration	Low-intensity laser therapy over nerve roots C5, C6, C7, and T1	It reduces pain and swelling and can progressively improve nerve function	Wavelength, 780 nm power, 250 mV. 450 J/mm^2^ for 20 mins
To regain neuromuscular control	Joint compression technique	To improve proprioception, joint stability, and muscle tone. It also enhances limb position awareness, improves motor function, and increases joint stability	10 repetitions with 30-seconds holds
	Lateralization training	Used for implicit motor imagery	5 to 10 minutes twice daily
	Rood’s facilitatory technique by using cotton balls (slow-light touch) and rotatory brush (fast brushing) on respective	This technique increases modulation of muscle spindle sensitivity to increase muscle activity and regains motor control	3 to 5 times followed by a 30-second break
	Sensory re-education with different textures such as sandpaper, silk, and net	For the re-establishment of impaired afferent sensory pathways or to restore sensory integration	3 to 5 times followed by a 30-second break
	Motor re-education through PNF rhythmic initiation technique (active assisted D1 –D2 flexion/extension) and range of motion exercises of bilateral upper extremity	To stimulate and improve neuromuscular control and to restore voluntary and active range of motion	10 repetitions x 2 sets

**Figure 6:**
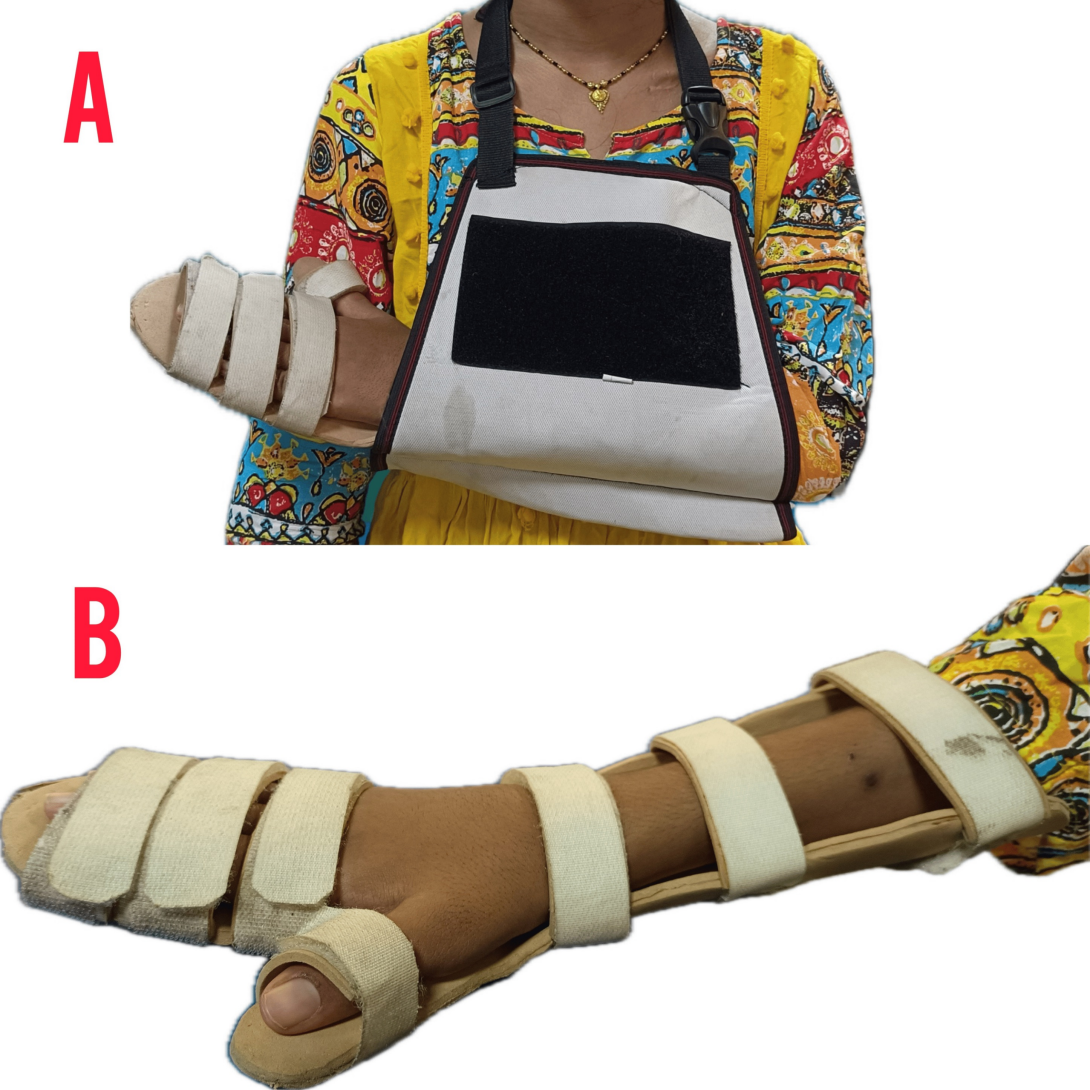
Immobilization in hemi-sling via Velpeau bandaging

Immobilization in a hemi-sling using Velpeau bandaging involves positioning the injured arm across the chest with the elbow bent at a 90-degree angle. A triangular bandage is placed over the arm, with the ends tied behind the neck to support the arm. A roller bandage is then applied in a figure-of-eight pattern, starting at the elbow, wrapping around the back to the opposite shoulder, and then around the chest, ensuring each layer overlaps. The bandage is secured with a safety pin or tape, ensuring the arm is snugly but comfortably immobilized, with regular checks for proper circulation as seen in Figure 6. 

In phase 2 (four to eight weeks) of the therapeutic intervention for a patient, the focus shifts to enhancing muscle strength, endurance, and functional mobility. Building on the foundational improvements from Phase 1, more active and resistive exercises are introduced. Techniques such as dynamic reversals, where the patient actively moves through a range of motion against resistance, and contract-relax methods to improve flexibility and muscle activation are emphasized. Weight-bearing exercises and more complex movement patterns are gradually incorporated to promote functional strength and stability. The intensity of exercises is increased, while still ensuring proper technique and patient safety. The goal is to progress towards more functional activities, preparing the patient for advanced phases of rehabilitation that target specific daily tasks and overall independence (Table 3). 

**Table 3:** Phase 2 (4-8 weeks) of therapeutic intervention provided to the patient* *[15]

Goals	Intervention	Rationale	Dosage
To enhance recovery of motor function: retard muscle weakness/wasting and promote muscle re-education	Electrical stimulation, long duration 300 ms interrupted galvanic (IG) stimulation at each motor point	Used for motor relearning and to elicit contraction of targeted muscle/nerve. It also promotes nerve healing and regeneration	30 contractions at each motor point
Gradual restoration of upper extremity strength and endurance	Graded motor imagery technique includes imagination of movements without performing them and mirrors visual feedback	Used to facilitate motor control. It provides immediate feedback and facilitates re-learning through visual and auditory feedback	Each movement 10 times twice a day
Implement strategies for sensory and motor re-education	Sensory re-education via sensory kit along the dermatomal distribution (with different textures and shapes with eyes open and eyes closed)	For the re-establishment of impaired afferent sensory pathways or to restore sensory integration and to accelerate sensorimotor recovery	3 to 5 times followed by a 30-second break for each dermatome distribution
Restoration of functions as soon as neural regulation takes place	Roods facilitatory approach with (fast tapping over the muscle belly)	For facilitating and re-educating muscles to gain voluntary muscular contractions	10 repetitions x 2 sets

In Phase 3 (8-12 weeks) of the therapeutic intervention for a patient, the focus is on advanced strengthening, coordination, and functional training to further enhance the patient’s capabilities and independence. During this phase, exercises are more challenging and closely mimic daily activities. High-intensity resistance training, balance exercises, and advanced PNF patterns are employed to refine motor control and stability. Functional activities such as gait training, stair climbing, and simulated tasks relevant to the patient's lifestyle are integrated to promote the practical application of skills. The intensity and complexity of exercises are tailored to the patient's progress, ensuring continual improvement while minimizing the risk of injury. The goal is to achieve a high level of functional independence and prepare the patient for a return to normal daily activities (Table 4).

**Table 4:** Phase 3 (8-12 weeks) of therapeutic intervention provided to the patient* *[16]

Goals	Intervention	Rationale	Dosage
To regain strength and neuromuscular control, to facilitate motor re-education	AO-PNS (action observation with peripheral nerve stimulation) technique, demonstration of repetitive movement pattern while receiving electrical stimulation	To elicit motor re-education also electrical stimulation enhances axon growth during nerve repair and accelerates motor recovery	10 repetitions x 2 sets
Foster a proactive approach to health and wellness, incorporating regular exercise and preventive measures, for the gradual restoration of movements	Kinesiotherapy training in the gravity-eliminated or gravity-reduced positions, increasing training with light weights progressed to antigravity position	To maintain and promote range of motion, joint integrity, and strength. To rehabilitate and enhance mobility, endurance, and strength	10 repetitions x 2 sets
To promote strengthening	Isometrics contractions of shoulder flexion, external/internal rotations, adduction and abduction	To enhance muscle activation as well as promote strengthening	5 repetitions each with 30 seconds of hold

Outcomes

The outcome measure evaluation of the patient is shown in Table 5.

**Table 5:** Outcome measures: pre- and post-intervention VAS: visual analog scale; PROM: passive range of motion; MMT: manual muscle testing; BPOM: brachial plexus outcome measure; UEFS: Upper Extremity Functional Scale; MRC: Medical Research Council Normal ROM, shoulder flexion: 0-180°; shoulder extension: 0-180°; shoulder abduction: 0-180°; shoulder adduction: 180-0°

Outcomes	Day 1 assessment (pre-intervention)	Post-intervention (1 month)	Post-intervention (2 months)
VAS	On rest: 5/10	On rest: 4/10	On rest: 1/10
	On activity: 8/10	On activity: 6/10	On activity: 3/10
PROM			
Shoulder flexion	0-25°	0-50°	0-100°
Shoulder extension	0-10°	0-25°	0-35°
Shoulder abduction	0-30°	0-50°	0-80°
Shoulder adduction	30-0°	50-0°	80-0°
MMT			
Shoulder flexors	1+/5	2/5	2/5
Shoulder extensors	1/5	1+/5	2/5
Shoulder abductors	1/5	1+/5	2/5
Shoulder adductors	1/5	1+/5	2/5
BPOM	11/55	28/55	36/55
UEFS	20/100	40/100	58/100
MRC dyspnoea scale	4/5	1/5	0/5
Barthel Index	35/100	75/100	85/100

## Discussion

This case highlights the complexity of managing a displaced mid-shaft clavicular fracture with concurrent BPI following a road traffic accident. The multidisciplinary approach, including timely imaging, surgical intervention, and comprehensive physiotherapy, is crucial for optimal recovery and functional restoration. Clavicle fractures can rarely lead to compression of the brachial plexus. On the other hand, traumatic elongations of the brachial plexus are more commonly observed, often due to high-energy traumas. These elongations frequently coincide with fractures of the cervical spine, ribs, humerus, scapula, and clavicle.

Compression of the brachial plexus typically leads to neurapraxia without axonotmesis, resulting in secondary thoracic outlet syndrome that often requires surgical intervention. Symptoms may manifest as pain, weakness, rapid fatigue of shoulder girdle muscles, and numbness or paresthesia in the arm and fingers, exacerbated by repetitive shoulder movements. Surgical options for treating late neurologic symptomatic clavicle fractures include first rib resection, scalenectomy, clavicle resection, costoclavicular space decompression, and corrective osteotomy with internal fixation [17].

Gushikem et al. showed that the application of neurophysiotherapy serves to prevent the occurrence of joint stiffness, muscle atrophy, and contractures, while simultaneously reinstating the complete range of motion and improving the overall quality of life. Traumatic BPIs, commonly caused by road traffic crashes, often require surgical intervention for repair. These intricate injuries necessitate long-term and intensive therapeutic interventions and individuals affected by such injuries frequently face challenges in carrying out daily tasks and returning to work, particularly if their occupation involves manual labor [18]. In a similar study, Deodhe et al. heightened challenges in carrying out everyday activities as UE function declined. Following a six-week intervention involving PNF and functional electrical stimulation (FES), a notable enhancement in UE function was noted. Consequently, the PNF and FES proved to be beneficial in improving functional mobility after traumatic BPI [19]. Dave et al. found that the combination of electrical stimulation and PNF therapy was more successful in rehabilitating BPIs than individual therapies on their own [20].

## Conclusions

This report underscores the complexity and challenges of managing a displaced mid-shaft clavicular fracture with a concurrent BPI after a road traffic accident. The effective management of such injuries requires a multidisciplinary approach, including timely imaging, surgical intervention, and comprehensive physiotherapy. Early intervention helps to prevent secondary complications such as muscle atrophy and joint stiffness. This approach is crucial for optimal recovery and functional restoration. In rehabilitation programs, particularly those incorporating electrical stimulation and PNF techniques, graded motor imagery plays a vital role in improving functional mobility and overall quality of life. Early diagnosis, appropriate surgical intervention, and a structured rehabilitation plan are essential for ensuring the best functional outcomes and enhancing the patient's ability to return to daily activities and work.
